# Advanced systemic mastocytosis: the impact of KIT mutations in diagnosis, treatment, and progression

**DOI:** 10.1111/ejh.12043

**Published:** 2013-01-22

**Authors:** Srdan Verstovsek

**Affiliations:** Department of Leukemia, The University of Texas, MD Anderson Cancer CenterHouston, TX, USA

**Keywords:** advanced systemic mastocytosis, aggressive systemic mastocytosis, KIT, response criteria, tyrosine kinase inhibitor

## Abstract

Apart from indolent systemic mastocytosis (SM), which is associated with a favorable prognosis, other subtypes of SM (SM with associated clonal hematologic non–mast cell lineage disease, aggressive SM, and mast cell leukemia – collectively referred to in this review as advanced SM) can be debilitating. The complexity of SM makes both the diagnosis and design of response criteria challenging for clinical studies. The tyrosine kinase KIT has been shown to play a crucial role in the pathogenesis of SM and has been a focal point in the development of targeted therapy. Mutations within various domains of the KIT receptor that lead to constitutive activation have been identified in patients, and those involving the activation loop of the KIT receptor are the mutations most frequently detected in patients with mastocytosis. Aberrant activation of the KIT receptor results in increased production of mast cells in extracutaneous organs that may lead to organ failure or early death. This review discusses the diagnosis and management of patients with advanced SM, including the relevance of KIT in this disease, potential therapies targeting this kinase, and criteria for measuring responses to these therapies.

Mastocytosis, considered a subcategory of myeloproliferative neoplasms based on the World Health Organization criteria, is characterized by abnormal growth of mast cells (1). Categories of mastocytosis include cutaneous mastocytosis and systemic mastocytosis (SM). Cutaneous mastocytosis is the most frequent form of mast cell disease, has no organ involvement besides the skin, and is associated with a favorable prognosis (2, 3). SM involves mast cells infiltrating extracutaneous organs such as the bone marrow, spleen, and liver (1, 4). The clinical course of SM may range from benign [indolent SM (ISM)] to a more aggressive, life-threatening clinical course [aggressive SM (ASM), SM associated with clonal hematologic non–mast cell lineage disease (SM-AHNMD), and mast cell leukemia (MCL) – for the purposes of this review, collectively called ‘advanced SM’].

Most patients with SM have ISM. Although the survival of these patients is comparable to that of the general population, they can experience symptoms such as skin lesions, gastrointestinal symptoms, or mast cell mediator release symptoms, and patients with smoldering SM (vs. patients with any subtype of ISM) may have an increased risk of developing disease transformation to aggressive forms of SM (1). In contrast, the survival of patients with advanced SM is significantly shorter than that of the overall population and is affected by the disease subtype, with a median survival of 41 months for patients with ASM, 24 months for patients with SM-AHNMD, and 2 months for patients with MCL (5).

In patients with SM-AHNMD, prognosis can differ widely depending on the subgroup. In a study of 123 patients with SM-AHNMD, the SM-myeloproliferative neoplasm, SM-chronic myelomonocytic leukemia, SM-myelodysplastic syndrome, and SM-acute leukemia subgroups were associated with median survivals of 31, 15, 13, and 11 months, respectively (6). Patients with advanced SM may suffer from a multitude of disease-related symptoms and signs, such as anemia, thrombocytopenia, ascites, bone fractures, gastrointestinal abnormalities, and enlargement of the liver, spleen, and lymph nodes, which ultimately lead to organ failure and early death (7). No effective therapies exist for the majority of patients with advanced SM, but new treatments are being developed. Prominent among these are tyrosine kinase inhibitors (TKIs) targeting the KIT kinase.

## KIT as a diagnostic and prognostic marker in SM

**Figure 1:**
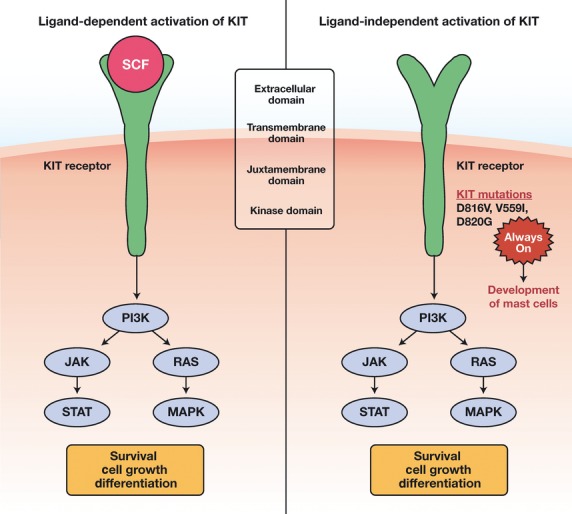
Depiction of ligand-dependent and ligand-independent activation of KIT caused by mutations (10, 56). JAK, Janus kinase; MAPK, mitogen-activated protein kinase; PI3K, phosphoinositide 3-kinase; SCF, stem cell factor; STAT, signal transducer and activator of transcription.

KIT is a receptor tyrosine kinase that plays a role in the proliferation of a number of cell types, including mast cells, melanocytes, germ cells, and hematopoietic stem cells (8, 9). KIT is normally activated upon binding of its ligand, stem cell factor, which triggers autophosphorylation and dimerization of KIT (8, 10). Once KIT is activated, downstream signaling through the phosphoinositide 3-kinase, Janus kinase/signal transducer and activator of transcription, and mitogen-activated protein kinase pathways induces cell proliferation and survival (Fig. 1) (8). The most common KIT mutation in patients with mastocytosis, aspartate to valine at residue 816 (D816V) (5), lies within the activation loop domain and causes a conformational change in the enzymatic pocket of the receptor. This conformational change results in ligand-independent constitutive activation of KIT and leads to increased proliferation and a reduction in apoptosis (5, 11, 12). In addition to activation loop mutations such as D816V, a number of other KIT mutations have been reported in patients with cutaneous mastocytosis, ISM, and advanced SM (Table 1). The presence of the D816V mutation is a minor criterion of diagnosis, but absence of the mutation does not exclude the diagnosis of SM (13, 14). Diagnosis of SM is a multi-step process, and all relevant criteria should be examined before a definitive diagnosis is established (Table 2).

**Table 1:** KIT-activating mutations in mastocytosis (56)

KIT mutation	Region of mutation	Mastocytosis disease(s) associated with mutation
A533D	Transmembrane domain	Familial cutaneous mastocytosis (57)
C443Y	Extracellular domain	Cutaneous pediatric mastocytosis (58)
D419Y	Extracellular domain	Cutaneous pediatric mastocytosis (58)
D572A	Juxtamembrane domain	Cutaneous pediatric mastocytosis (58)
D816F	Activation loop	SM (59)
D816H	Activation loop	SM and acute leukemia (60)
D816I	Activation loop	Cutaneous pediatric mastocytosis (58)
D816V	Activation loop	SM
		Cutaneous pediatric mastocytosis
		Familial cutaneous mastocytosis
		Aggressive SM (5, 57, 58, 61)
D816Y	Activation loop	SM (59)
D820G	Activation loop	Aggressive SM (62)
Del419	Extracellular domain	Familial cutaneous mastocytosis (26)
Dup(501–502)	Extracellular domain	Mast cell leukemia (44)
E839K	Activation loop	Urticarial pigmentosa (59)
F522C	Transmembrane domain	WDSM (26)
I817V	Activation loop	WDSM (15)
InsFF419	Extracellular domain	Cutaneous pediatric mastocytosis (58)
InsV815–I816	Activation loop	SM (15)
K509I	Extracellular domain	Familial SM (25)
N8221	Activation loop	Familial cutaneous mastocytosis (63)
R815K	Activation loop	Pediatric urticarial pigmentosa (64)
T417Y	Extracellular domain	Pediatric mastocytosis (58)
V560G	Juxtamembrane domain	SM Familial cutaneous mastocytosis (57, 65)
V559I	Juxtamembrane domain	Aggressive SM (66)
Y418Y	Extracellular domain	Cutaneous pediatric mastocytosis (58)

SM, systemic mastocytosis; WDSM, well-differentiated systemic mastocytosis.

**Table 2:** Diagnostic criteria for SM (67)

Disease state	Diagnostic criteria
SM	Presence of either 1 major and 1 minor or 3 minor criteria
	Major criteria
	•Multi-focal, dense mast cell infiltration (>15 mast cells in aggregates) in samples of BM and/or extracutaneous organs
	Minor criteria
	•Presence of D816V KIT mutation in bone marrow, blood, or extracutaneous tissues
	•Baseline serum tryptase concentration of >20 ng/mL
	•Expression of KIT plus CD2 and/or CD25 in mast cells from bone marrow
	•>25% of mast cells with atypical or spindle shape
SM-AHNMD	Meets criteria for SM and criteria for an associated clonal hematologic, non–mast cell lineage disease
ASM	Meets criteria for SM and has ≥1 C-finding
	C-findings
	•Low ANC, anemia (hemoglobin <10 g/dL), or thrombocytopenia (platelets <100 000/μL)
	•Hepatomegaly, ascites, impaired liver function, and/or portal hypertension
	•Malabsorption and weight loss
	•Osteolysis and/or osteoporosis
	•Splenomegaly
Mast cell leukemia	•BM biopsy with a diffuse infiltration of compact, immature, atypical mast cells
	•Aspirate smears with >20% mast cells
	•Peripheral blood
	◦Typical MCL: >10% mast cells
	◦Aleukemic MCL: <10% mast cells

ANC, absolute neutrophil count; ASM, aggressive SM; BM, bone marrow; MCL, mast cell leukemia; SM, systemic mastocytosis; SM-AHNMD, SM with a clonal hematologic non–mast cell lineage disease.

The D816V KIT mutation has been associated with higher bone marrow mast cell burden and the presence of C-findings, which are a measure of disease aggressiveness (5). Data from the prospective study by the Spanish Network on Mastocytosis (REMA) demonstrated that 81% of patients with advanced SM expressed D816V KIT in ≥2 bone marrow myeloid cell populations, compared with 27% of patients with good-prognosis SM, including ISM (15). Consistent with these findings, the long-term REMA study of 145 patients with ISM demonstrated that the presence of the D816V KIT mutation in all hematopoietic lineages and elevated β2-microglobulin levels was predictive of evolution to a more aggressive form of SM (16). Together, these reports suggest that multi-lineage D816V KIT is associated with more aggressive forms of mastocytosis.

It should be noted that the detection of a KIT mutation can depend both on assay sensitivity and on sample type. For example, KIT mutations are more easily detected in cells from the bone marrow compared with those isolated from peripheral blood. Currently, the most sensitive detection methods are real-time reverse-transcription polymerase chain reaction (RT-PCR) plus restriction fragment length polymorphism analysis, peptide nucleic acid–mediated PCR, and allele-specific PCR; any of these three techniques is recommended for assessing KIT mutations (17). In addition, detection of KIT mutations can be difficult when using fluorescence-activated cell sorting in samples not enriched for mast cells; thus, using unsorted bone marrow cells or cells from the peripheral blood of patients with a low mast cell burden may result in a higher rate of false-negative mutations in KIT (15). Because mast cell enrichment is not currently standard in clinical practice, a negative KIT mutation finding should be confirmed, particularly in patients with ASM (17).

## Response criteria in ASM

Aggressive SM is a complex and varied disease with unique manifestations in individual patients. To compare interventions and improve patient management, Valent *et al*. (7) developed response criteria for ASM, which they termed C-findings, based on the most common symptoms of this disease (Table 3). The criteria established by Valent *et al*. are the most commonly used standards for measuring response in patients treated for ASM (Table 4). These response measurements include the infiltration of mast cells in organs, tryptase levels, organomegaly, and C-findings (7). A major response is defined as having ≥1 C-finding completely resolved, with no progression in other C-findings; a disappearance or decrease of mast cell infiltration in organs and organomegaly; and a decrease of serum tryptase levels to <20 ng/mL. A partial response is defined as having ≥1 C-finding that decreases by >50% (good partial response) or decreases by ≤50% (minor response) and no increase in any other C-findings. No response is defined as having no change or an increase in C-findings (Table 4) (7).

**Table 3:** Measurable C-findings for ASM (7, 67)

C-Findings for ASM	MR	GPR
Anemia (Hb <10 g/dL)	Hb >10 g/dL	↓ <10 g/dL, reverted by >50%
Thrombocytopenia (platelets <100 000/μL)	Platelets >100 000/μL	↓ <100 000/μL, reverted by >50%
ANC ≤ 1000/μL	ANC > 1000/μL	↓ 1000/μL, reverted by >50%
Hepatopathy	Returned to normal levels	Reverted by >50%
Splenomegaly	No signs of hypersplenism	Hypersplenism improved by >50%
Malabsorption with hypoalbuminemia and/or weight loss	Normal albumin and weight	Albumin level improved by >50% Weight loss reverted by >50% or regaining >5% of weight

ANC, absolute neutrophil count; ASM, aggressive systemic mastocytosis; GPR, good partial response; Hb, hemoglobin; MR, major response.

**Table 4:** Aggressive systemic mastocytosis response criteria developed by Valent *et al*. (7)

Response	CF	Subcategory	MC infiltrate in organ	Tryptase level	Organomegaly
MR	≥1 CF resolved and no CF ↑	CR	MC infiltrate disappeared and ↓ tryptase <20 ng/mL and organomegaly disappeared		
	≥1 CF resolved and no CF ↑	IR	MC infiltrate decreased and/or visible regression of organomegaly		
	≥1 CF resolved and no CF ↑	PCR	No significant change	↓ ≤50% to 0%	No significant change
PR	≥1 CF ↓ by >50%; no CF ↑	GPR	N/A	N/A	No significant change
	≥1 CF ↓ by ≤50%; no CF ↑	MinR	N/A	N/A	No significant change
NR	CFs show constant range	SD	N/A	N/A	N/A
	≥1 CF show progression	PD	N/A	N/A	N/A

CF, C-finding; CR, complete remission; GPR, good partial response; IR, incomplete remission; MC, mast cell; MinR, minor response; MR, major response; N/A, not applicable; NR, no response; PCR, pure clinical response; PD, progressive disease; PR, partial response; SD, stable disease.

Although the Valent criteria are the most widely used for evaluating response, they do not provide a complete assessment for all patients with ASM. For instance, some patients may have >1 C-finding resolved but may require red blood cell and/or platelet transfusions. Furthermore, they do not account for other important factors, such as the minimum duration of an improvement needed to qualify as a response or the overall duration of response to treatment. Experts from the Mayo Clinic published new recommendations to make response criteria more intuitive, standard, objective, and reproducible for practicing physicians (Table 5) (18). In addition, they suggested that the response criteria for SM-AHNMD should follow the treatment response criteria for AHNMD and not SM and that MCL treatment response criteria should follow the response criteria for acute leukemia (18). This view is not shared by Valent *et al*. (19), who argued that MCL is clinically, histologically, and genetically more similar to ASM than to acute leukemia. These issues remain a topic of active debate.

**Table 5:** Proposed response criteria for ASM according to Pardanani and Tefferi (18)

Response category	A: disease-related symptoms^1^	B: organomegaly/lymphadenopathy^2^	C: disease-related organopathy^3^	D: BM findings^4^
Complete response: A + B + C + D required (when present)	Complete resolution for 3 months	Complete resolution^2^	Complete resolution^5^	Absence of abnormal MC infiltration^6^
Major response: A + B + C + D required (when present)	No progression (at a minimum)	No progression (at a minimum)	Complete resolution of ≥1 element of organopathy^3^,^7^	>50% Decrease in BM MC (%)
Partial response: A or B or C (without progression in others)	Complete resolution for 3 months	Complete resolution^2^	≥2 Grade improvement in ≥1 element of organopathy^7^,^8^	No progression (at a minimum)
Stable disease	None of the above responses			
Progressive disease: B or C required	Not applicable^9^	>50% Increase from baseline^2^	≥2 Grade worsening from baseline	Not applicable

ASM, aggressive systemic mastocytosis; BM, bone marrow; MC, mast cell.

Responses were validated only if they lasted for ≥4 wk.

1 To be considered as a parameter for response measurement, symptoms must be frequent (≥1 time per month); severe enough to require treatment, despite prophylaxis (H1 and H2 histamine receptor antagonists, proton pump inhibitors, and/or oral cromolyn sodium); and accompanied by either organomegaly/lymphadenopathy or organopathy.

2 Palpable disease or measurable disease by imaging studies required at baseline; baseline and post-treatment status must be documented by imaging studies to allow third-party confirmation of response or progression.

3 Grade ≥2 ascites (not optimally controlled with medical therapy) or grade ≥2 weight loss or grade ≥2 osteoporosis (large osteolytic lesions or pathological fracture) or grade ≥2 anemia (hemoglobin <10 g/dL) or thrombocytopenia (platelet count <75 × 10^9^/L) or grade ≥2 hyperbilirubinemia or hypoalbuminemia that is a disease-related change from baseline (grades are per National Cancer Institute Common Toxicity Criteria for reporting adverse events).

4 BM characteristics to be described: (i) BM MC burden (%) based on tryptase/CD117 (KIT) immunostaining, (ii) cytogenetics, and (iii) D816V KIT status.

5 Complete resolution of all evidence of organopathy unless observed changes are deemed related to treatment.

6 Cytogenetic remission is not required; cytogenetic response, if any, to be documented as follows: complete response, disappearance of previously documented chromosomal abnormality without appearance of new ones and partial response, ≥50% reduction of cytogenetic abnormality.

7 No progression in other elements of organopathy should be evident unless observed changes are deemed related to treatment.

8 Per National Cancer Institute Common Toxicity Criteria for reporting adverse events.

9 Given the difficulty in distinguishing treatment-related symptoms from disease-related symptoms.

With such variations, the debate over the optimal response criteria to use when evaluating drug efficacy in patients with advanced SM will likely continue. The response criteria used in clinical trials of TKIs such as nilotinib, imatinib, midostaurin, and dasatinib have differed among studies (Table 6). These non-standardized response criteria and the small patient populations in many of the trials have made it difficult to determine the effectiveness of available therapeutic agents and compare their benefits. Therefore, the current movement toward standardization is critical to establish consensus response criteria for patients with advanced SM.

**Table 6:** Clinical studies of tyrosine kinase inhibitors in patients with SM

TKI drug	Population	*N*	% of Patients investigated with D816V KIT mutation	Criteria for response	Responses
Nilotinib (35)	SM	60	30/36 (83%)	Serum tryptase	3% CR,^1^ 8% IR, 7% MinR, <1% PR
				Bone marrow mast cell counts	
				Improvement of clinical symptoms	
Imatinib (68)	SM	22	86%	Slightly modified from Valent response criteria	18% ORR (50% of ASM); 9% MR, 9% PR
Midostaurin (47)	SM [4 SM, 14 SM-CMML, 4 SM-MDS (MPN-U in 1), and 4 MCL]	26	69%	Valent response criteria	69% ORR; 38% MR (23% IR, 15% PCR), 31% PR (19% GPR, 12% MinR), 15% SD, 15% PD
Dasatinib (39)	SM (9 ASM, 18 ISM, and 6 SM-AHNMD)	33	85%	Valent response criteria	33% ORR; 2 CR in SM-PMF, SM-CEL (KIT D816V negative); 9 with symptomatic improvement (6 ISM, 3 ASM; 8 of 9 were KIT D816V positive)
Dasatinib (41)	SM (2 ASM, 1 ISM, and 1 SM-AHNMD)	4	100%	Major resolution of symptoms, C-finding responses	50% Major resolution of diarrhea, pruritus (1 patient with ASM); 25% major resolution of rash; 25% major C-finding response (weight gain, patient with ASM)

ASM, aggressive SM; CM, cutaneous mastocytosis; CR, complete remission; GPR, good partial response; IR, incomplete remission; ISM, indolent SM; MCL, mast cell leukemia; MinR, minor response; MPN-U, myeloproliferative neoplasm, unclassifiable; MR, major response; ORR, overall response rate; PCR, pure clinical response; PD, progressive disease; PR, partial response; SD, stable disease; SM, systemic mastocytosis; SM-AHNMD, SM with clonal hematologic non–mast cell lineage disease; SM-CEL, SM–chronic eosinophilic leukemia; SM-CMML, SM–chronic myelomonocytic leukemia; SM-MDS, SM–myelodysplastic syndrome; SM-PMF, SM–primary myelofibrosis; TKI, tyrosine kinase inhibitor.

1 Defined by investigator assessment of bone marrow mast cell counts, serum tryptase levels, and improvement in clinical symptoms.

## Current and potential KIT-targeted therapies in advanced SM

There is no standard treatment regimen for advanced SM. Interferon α (IFN-α), cladribine, and hydroxyurea (HU) have been used to manage patients with advanced SM. A retrospective study examined the efficacy of these agents in patients with ISM, ASM, SM-AHNMD, or MCL (20). This analysis defined responses according to modified Valent criteria. Overall response rate (ORR) was defined as the sum of complete responses (all clinical symptoms and signs were completely resolved for ≥1 month), major responses (>50% improvement in symptoms and signs), and partial responses (10–50% improvement in symptoms and signs). The ORR among evaluable patients with SM treated with IFN-α (*n* = 40) was 53%, and patients had a median duration of response of 12 months. ORR for patients treated with cladribine (*n* = 22) was 55%, with a median duration of response of 11 months. Patients treated with HU had a much poorer response to treatment, with an ORR of 19% and a median duration of response of 31.5 months (20). Because the Mayo Clinic study population consisted of a heterogeneous group of patients, including those with indolent and aggressive SM, these data may not accurately reflect the rates and duration of responses in patients with ASM.

Overall, these therapies provided symptomatic relief but did not substantially affect mast cell burden. Newer evidence-based approaches to the treatment of advanced SM have focused on KIT inhibitors because of the ubiquity of KIT mutations in patients with advanced SM and the importance of this protein in normal mast cell function.

### Imatinib

The primary targets of the TKI imatinib include BCR-ABL, KIT, and platelet-derived growth factor receptor (PDGFR) (21). Imatinib inhibits wild-type (WT) KIT and the V560G KIT mutant (12). However, imatinib is less effective against the D816V KIT mutation *in vitro* (12, 22) and in the clinic (23, 24). Currently, imatinib is the only therapeutic agent approved for patients with SM, specifically for adult patients with ASM without the D816V mutation or with unknown KIT mutational status. Food and Drug Administration approval was based on data in five patients with ASM from a phase 2 study and several case studies demonstrating that imatinib was effective in treating patients with ASM with KIT mutations other than D816V, including F522C and K509I (25–27). The approval for patients with ‘unknown’ D816V mutation status at least in part lies in the difficulties one faces in getting proper testing carried out in everyday practice, as discussed above. All patients with the FIP1 like 1–PDGFRα fusion kinase, which is present in some patients with hypereosinophilic syndrome (28) and correlates with response to imatinib (29), achieved a complete hematologic response. All patients with a juxtamembrane KIT mutation achieved a partial hematologic response (27). In addition, 44% of patients with WT KIT or with an unknown mutation achieved a partial hematologic response. Four patients harbored the D816V KIT mutation; of these patients, only one (who had concomitant chronic myeloid leukemia and ASM) achieved a hematologic response (27).

Patients who are diagnosed with well-differentiated SM (WDSM) may also benefit from imatinib treatment because WDSM is associated with lower frequencies of KIT mutations involving the activation loop, such as the imatinib-resistant D816V KIT mutation, compared with patients with advanced SM (26). Treatment with imatinib reduced mast cell infiltration into bone marrow and resolved symptoms in a patient with WDSM (26), and data from a recent case report demonstrated that a patient with WDSM achieved a complete response on imatinib beyond 1 yr (30), suggesting that imatinib may be associated with a therapeutic benefit for patients with WDSM.

### Nilotinib

Nilotinib is a TKI that also inhibits BCR-ABL, PDGFR, and KIT (31, 32). Low concentrations of nilotinib reduced the growth of and induced apoptosis in transformed murine Ba/F3 cells expressing the D814V KIT mutation, which corresponds to the human D816V KIT mutation (33). However, nilotinib was not sufficient to inhibit the growth of D816V KIT–positive bone marrow mast cells obtained from patients with ASM, but showed activity in cell lines expressing WT KIT and the very rare D560G KIT mutation (34). In a phase 2 clinical study, an ORR of 20% (12 of 60) was seen in patients with SM (83% D816V KIT positive) treated with nilotinib and included two complete responses, one partial response, five incomplete responses, and four minor responses (35). The toxicity profile with nilotinib was consistent with that seen in other studies (36). Grade 3/4 adverse events included headache in three patients (5%), diarrhea in four patients (7%), and thrombocytopenia in three patients (5%) (35).

### Dasatinib

Dasatinib is an inhibitor that targets BCR-ABL, SRC, and other tyrosine kinases, including KIT (37). Dasatinib has activity *in vitro* against both WT and D816V KIT (38). In a phase 2 study, the ORR in patients with SM (the majority of whom had the D816V mutation) was 33% (11 of 33). Both patients who achieved a complete response were negative for D816V KIT (39). The most common grade 3 non-hematologic adverse events included headache (12%), pain (9% of 33), pleural effusion (6%), and dyspnea (6%), which are consistent with other reports (40). Grade 3 hematologic toxicity occurred in two patients (anemia and thrombocytopenia in one patient each); no grade 4 toxicities were observed (39). Several other case reports have documented anecdotal efficacy of dasatinib in controlling symptoms in patients with SM, including a series of four patients treated with dasatinib who experienced major resolutions of diarrhea, pruritus, rash, and weight gain (41). A phase 2 study of dasatinib in patients with SM is planned (NCT00979160).

### Masitinib

Masitinib is a multi-targeted inhibitor of the KIT, PDGFR, fibroblast growth factor receptor 3, and Lyn tyrosine kinases. An *in vitro* assay has shown that masitinib inhibits WT KIT (IC_50_ 200 nm) with greater potency compared with D816V KIT (IC_50_ 5.0 μm) (42) and has demonstrated activity in a phase II study of patients with systemic or cutaneous mastocytosis who had at least two organs confirmed to have mast cell infiltration, one or both of which had to have no detectable mutations in KIT (43). There were 25 patients in this study (without a detectable D816V mutation), and a clinical response was observed in 56% of patients. Adverse events were generally mild to moderate and occurred early after initiation of masitinib treatment. The rates of response to masitinib were similar in patients with KIT mutation in one infiltrated organ and patients with no KIT mutations in any infiltrated organs, suggesting that masitinib may have clinical activity in patients harboring KIT mutations. Consistent with this report, data from a recent case study demonstrated that a patient with SM who developed the KIT mutation dup(501–503) prior to therapy had clinical improvement, with disappearance of circulating mast cells and decreased serum histamine and tryptase levels (44). A phase 3 study of masitinib in patients with smoldering SM, ISM, or cutaneous mastocytosis is currently recruiting (NCT00814073).

### Midostaurin

Midostaurin is an inhibitor of several tyrosine kinases including KIT, fms-related tyrosine kinase 3, vascular endothelial growth factor receptor 2, and PDGFR (45, 46). *In vitro* studies have demonstrated that midostaurin is effective in inhibiting both WT and D816V KIT (45–47). In addition, midostaurin can act synergistically with nilotinib to inhibit the growth of mast cells with the D816V mutation (48). Initial results from a phase 2 study of single-agent midostaurin showed promising activity in 26 patients with advanced SM, with a 69% response rate: 38% major response, 19% good partial response, and 12% minor partial response (47). Importantly, all patients who achieved a major response had D816V KIT, and the response was durable for a median of 1.5 yr. In responding patients, midostaurin improved organ function while alleviating mediator-related symptoms, reduced serum tryptase levels by >50%, reduced splenomegaly, and reduced mast cell burden in the bone marrow. Midostaurin was relatively well tolerated. Grade 3/4 adverse events included nausea (4%), fatigue (4%), increased lipase (4%), rash (4%), anemia (17%), neutropenia (4%), and thrombocytopenia (4%) (47).

Similarly, in a compassionate use program in the United Kingdom, two of 10 evaluable patients with SM who were treated with midostaurin experienced major regression, four patients showed partial regression, and three patients achieved continuous complete regression for >2 yr (49). A larger phase 2 clinical trial in patients with ASM or MCL is ongoing (NCT00782067). Results from this study, presented at the 2012 American Society of Hematology Annual Meeting, demonstrated that midostaurin was associated with durable responses, with an ORR of 60% (53% major response) in 40 evaluable patients. Median duration of response and median overall survival have not been reached with 27-month median follow-up (50).

## Conclusions

Deregulation of KIT, most commonly through the D816V mutation (51), is the most frequent molecular abnormality observed in advanced SM (9). However, a number of other pathways have also been shown to play a role in the pathobiology of mastocytosis, including those downstream of PDGFR (52), basic fibroblast growth factor (53), transforming growth factor β (53), and the RAS pathway (54). Thus, one may speculate that a multi-targeted approach to the treatment of advanced SM may provide improved clinical efficacy over KIT TKI monotherapy alone. Systemic agents such as HU and IFN-α have had modest activity in patients with advanced SM and function primarily to manage symptoms.

The multi-targeted TKI midostaurin may decrease mast cell burden and has demonstrated sustained activity in some of these patients. A phase II study of midostaurin in patients diagnosed with advanced SM is currently underway to confirm these early results in a larger group of patients, and preliminary data from this trial are promising (50). Looking ahead to the possibility of a combination approach, ponatinib, an inhibitor of BCR-ABL and KIT, and midostaurin inhibited cell growth and induced apoptosis in the human MCL cell line HMC-1 when administered together (55). Further efforts are needed to standardize response criteria and reporting for this complex and diverse disease to properly evaluate the results of clinical trials in ASM and for clinicians to make informed decisions about the best treatment options for their patients.
